# Specialized Microbiome of a Halophyte and its Role in Helping Non-Host Plants to Withstand Salinity

**DOI:** 10.1038/srep32467

**Published:** 2016-08-30

**Authors:** Zhilin Yuan, Irina S. Druzhinina, Jessy Labbé, Regina Redman, Yuan Qin, Russell Rodriguez, Chulong Zhang, Gerald A. Tuskan, Fucheng Lin

**Affiliations:** 1 Institute of Subtropical Forestry, Chinese Academy of Forestry, Hangzhou, P. R. China; 2 Research Area Biochemical Technology, Institute of Chemical Engineering, TU Wien, Vienna, Austria; 3 Biosciences Division, Oak Ridge National Laboratory, Oak Ridge, USA; 4 Adaptive Symbiotic Technologies, Seattle, USA; 5 Depart of Biology, University of Washington, Seattle, USA; 6 State Key Laboratory for Rice Biology, Institute of Biotechnology, Zhejiang University, P. R. China

## Abstract

Root microbiota is a crucial determinant of plant productivity and stress tolerance. Here, we hypothesize that the superior halo-tolerance of seepweed *Suaeda salsa* is tightly linked to a specialized belowground microbiome. To test this hypothesis, we performed a phylogenetic trait-based framework analysis based on bacterial 16S rRNA gene and fungal nuclear rRNA internal transcribed spacer profiling. Data showed that the dominant α-proteobacteria and γ-proteobacteria communities in bulk soil and root endosphere tend to be phylogenetically clustered and at the same time exhibit phylogenetic over-dispersion in rhizosphere. Likewise, the dominant fungal genera occurred at high phylogenetic redundancy. Interestingly, we found the genomes of rhizospheric and endophytic bacteria associated with *S. salsa* to be enriched in genes contributing to salt stress acclimatization, nutrient solubilization and competitive root colonization. A wide diversity of rhizobacteria with similarity to known halotolerant taxa further supported this interpretation. These findings suggest that an ecological patterned root-microbial interaction strategy has been adopted in *S. salsa* system to confront soil salinity. We also demonstrated that the potential core microbiome members improve non-host plants growth and salt tolerance. This work provides a platform to improve plant fitness with halophytes-microbial associates and novel insights into the functions of plant microbiome under salinity.

Increasing soil salinity is an environmental problem that challenges agriculture worldwide^1^. Enhancing plant tolerance to salt merits extensive investigation as it will not only expand our understanding of the basic physiology and evolution of plants, but will also facilitate the improvement of crop production and the rehabilitation of saline soils. To date, a tremendous amount of fundamental research has been exclusively focused on characterizing an array of salt stress-related genes in plants with an applied effort to improve plant salt tolerance using genetic modification approaches. However, there has been only minor success from these approaches as such investigations often overlook the microbial contributions to plant ecophysiology^1^.

A recent eco-physiological approach suggests that the plant-associated microbial community may be the key factor for understanding the adaptation of plants to their habitat^2^. One intriguing example is the phenomenon of habitat-adapted symbiosis^3^, which means that plant adaptation to adverse environments is often achieved by forming symbiotic associations with non-mycorrhizal fungal endophytes. This type of symbiotically conferred plant stress tolerance typically occurs in a habitat-specific manner. The authors found that the endophytes *Curvularia protuberata* Cp4666D (Pleosporales) isolated from plants in in geothermal soils and *Fusarium culmorum* FcRed1 (Hypocreales) isolated from plants in coastal saline soils show potential for commercialization with proof of crop heat and salt tolerance enhancement, respectively^2^,^3^. Rather than isolating individual microbial species, a mixture of endophytic fungi and bacteria inhabiting the seeds of desert plants was transferred into crops and it was found that these microbes could confer similar beneficial functions in crops as in desert plants^4^. These striking findings imply that microbial-mediated plant traits not only rely on individual members in a community, but also on the cooperation and the functions of the entire microbiome^5^,^6^,^7^,^8^. These studies have had a profound impact on the development of biofertilizers, effectively shifting the focus from plant-microbe to plant-microbiome interactions. Numerous recent reports have highlighted and underscored the influence of the total soil microbiome on plant metabolism^9^, drought tolerance^10^,^11^ and even flowering phenology^12^.

There are now clear evidences that microorganisms found in association with plants growing in harsh environmental conditions help them to gain tolerance to abiotic stresses^3^,^4^,^10^,^13^,^14^,^15^. Therefore, such microorganisms are now being developed as biofertilizers^3^. In this context, we speculate that a better understanding of the microbiomes from saline environments, in particular in the rhizo- and endosphere of naturally occurring plants, will likely open up a new avenue of understanding plant salt resistance and how it is influenced by associated microorganisms^16^. While a number of studies dealt with the structure of microbial associates in halophytes and their potential phytobeneficial effects^17^,^18^,^19^,^20^, very little is known about the mechanisms by which halophyte-associated bacterial and fungal microbiome adapt to extreme salinity and how do they influence the plant phenotype.

The main hypothesis of this study was that the native superior halo-tolerant coastal plant *Suaeda salsa* (Amaranthaceae) has habitat-specific belowground microbial communities, which likely possess evolutionarily adaptive traits responding to high salt environments. We also assumed that microorganisms associated with the rhizosphere and endosphere of *S. salsa* may be beneficial for other plants, ultimately including agricultural crops. Consequently, the purpose of this work was to investigate the assembly and structure of *in situ* bacterial and fungal microbiomes associated with root endosphere and rhizosphere. We focused on measuring the multiple microbial traits related to salt adaptation and estimation of functional extensions of plant phenotypes in a phylogenetic framework. We further adapted a bottom-up approach to demonstrate the phytobeneficial effects of the core culturable microorganisms on stress tolerance in agricultural crops.

## Results

### α-and γ-proteobacteria: the belowground microbiome of *S. salsa*

We measured the phylogenetic diversity and structure of the bacterial communities at the class level. As the two classes, α-proteobacteria and γ-proteobacteria, represented the primary members of the belowground microbiome within amplicons (see Supplementary Figs S1 and S2). The α-proteobacteria and γ-proteobacteria communities in the root samples had the lowest Faith’s PD and Rao’s quadratic entropy (QE) values, two commonly used phylogenetic diversity indices, suggesting that there were comparatively higher levels of phylogenetic diversity in the bulk and rhizosphere soil than in the root endosphere (Table 1). The negative values for both the Net Relatedness Index (NRI) and the Nearest Taxon Index (NTI), two indices used to quantify the phylogenetic community structure, in the rhizosphere-associated α-proteobacteria and γ-proteobacteria microbiomes indicated a pattern of phylogenetic over-dispersion; this pattern became stronger and statistically significant as indicated by NRI (p ≤ 0.001). On the contrary, the bulk soil and root endosphere communities tended to be more phylogenetically clustered, although the p-values for NRI/NTI were not all significant for these communities (Table 1).

### Diverse halotolerant bacterial groups

**Figure 1:**
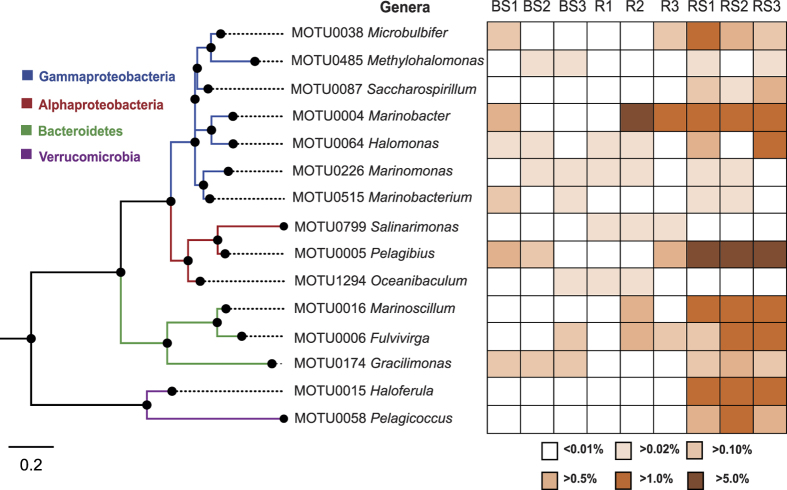
Phylogeny of diverse well-known halotolerant bacterial MOTUs found in belowground microbiome of *S. salsa* (classified into four classes: γ-proteobacteria, α-proteobacteria, Bacteroidetes and Verrucomicrobia) and their relative abundance in the root endosphere, rhizosphere soil and bulk soil habitats (R = root endosphere, RS = rhizosphere soil, BS = bulk soil).

At least fourteen bacterial MOTUs, showing strong phylogenetic affinities to well-known halotolerant species, were found across all samples. They fell into four phyla: δ-proteobacteria, α-proteobacteria, Bacteroidetes and Verrucomicrobia (Fig. 1). Within the soil rhizosphere, such genera as *Microbulbifer* (*Alteromonadales*), *Pelagibius* (*Rhodospirillales*), *Halomonas* (*Oceanospirillales*), *Marinoscillum* (*Sphingobacteriales*), *Fulvivirga* (*Flexibacteraceae*), *Haloferula* (*Verrucomicrobiales*), *Pelagicoccus* (*Puniceicoccales*) and *Marinobacter* (*Alteromonadales*) were exclusively enriched, with the exception that *Marinobacter* was more abundant in the root endosphere than in the bulk or rhizosphere soil.

### The *S. salsa* mycobiome, a diverse infrageneric community

**Figure 2:**
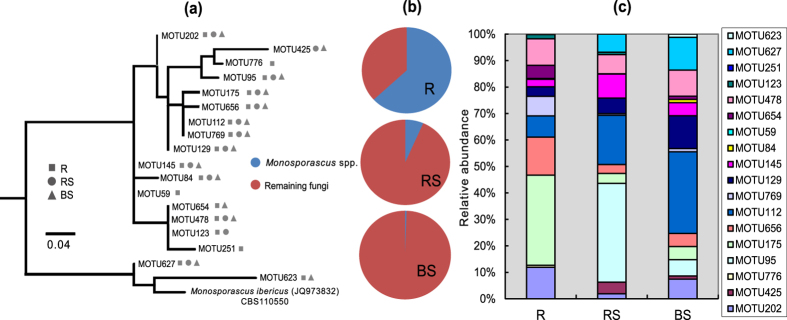
An unrooted phylogenetic tree of closely related MOTUs based on ITS1 sequences showing the infrageneric genetic diversity within *Monosporascus* genus. Percentages of the total *Monosporascus* members in the BS, RS and R habitats are indicated with pie charts. The relative abundance of each MOTU is presented with bar charts. R = root endosphere, RS = rhizosphere soil, BS = bulk soil.

We found several fungal genera that were represented by numerous ITS rRNA phylotypes, suggesting the sympatric presence of several closely related taxa from these genera in *S. salsa* belowground mycobiome (Table 2). Briefly, multiple MOTUs attributed to the genera *Beauveria* (Hypocreales, Ascomycota) and *Monosporascus* (Sordariales, Ascomycota) were heavily enriched in the bulk soil and root endosphere, respectively. Similarly, an array of MOTUs corresponding to the genera *Leptosphaeria* (Pleosporales, Ascomycota) and *Retroconis* (Pezizomycotina, Ascomycota) dominated the rhizosphere. All of the fungi displayed a moderate to high level of infrageneric diversity as revealed based on the calculation of mean p-distance and nucleotide diversity (Table 2). The highest mean p-distance value and the highest numbers of polymorphic sites were found in *Retroconis*, followed by *Leptosphaeria* and *Monosporascus* (Table 2), possibly signifying phylogenetic redundancy among preponderant fungal genera. We constructed a phylogeny of *Monosporascus* spp. based on their ITS1 rRNA sequences, as well as one sequence from the relevant species *M. ibericus* as an example to demonstrate infrageneric genetic distance and the relative abundance of each MOTU in the root endosphere, rhizosphere and bulk soils (Fig. 2a,c). It was shown that MOTUs affiliated to *Monosporascus* can be further divided into three main clades (Fig. 2a) and most of them were overlapped within the three ecological niches.

### Functional profiling of bacterial communities

We used the recent developed PICRUSt software tool to predict the functions without the shotgun metagenome analysis and to obtain a functional profile of bacterial microbiome. Mean (±standard deviation) weighted Nearest Sequenced Taxon Index (NSTI) values were 0.13 ± 0.02, 0.11 ± 0.01 and 0.28 ± 0.01 for bulk soil, rhizosphere soil and root endosphere, respectively. NSTI with our soil-based samples were lower than that reported for soil communities (0.17 ± 0.02), indicating that these samples provided an appropriate data set for accurate predictions, while root samples may have moderate or poor predictions owing to their relatively high NSTI values.

**Figure 3:**
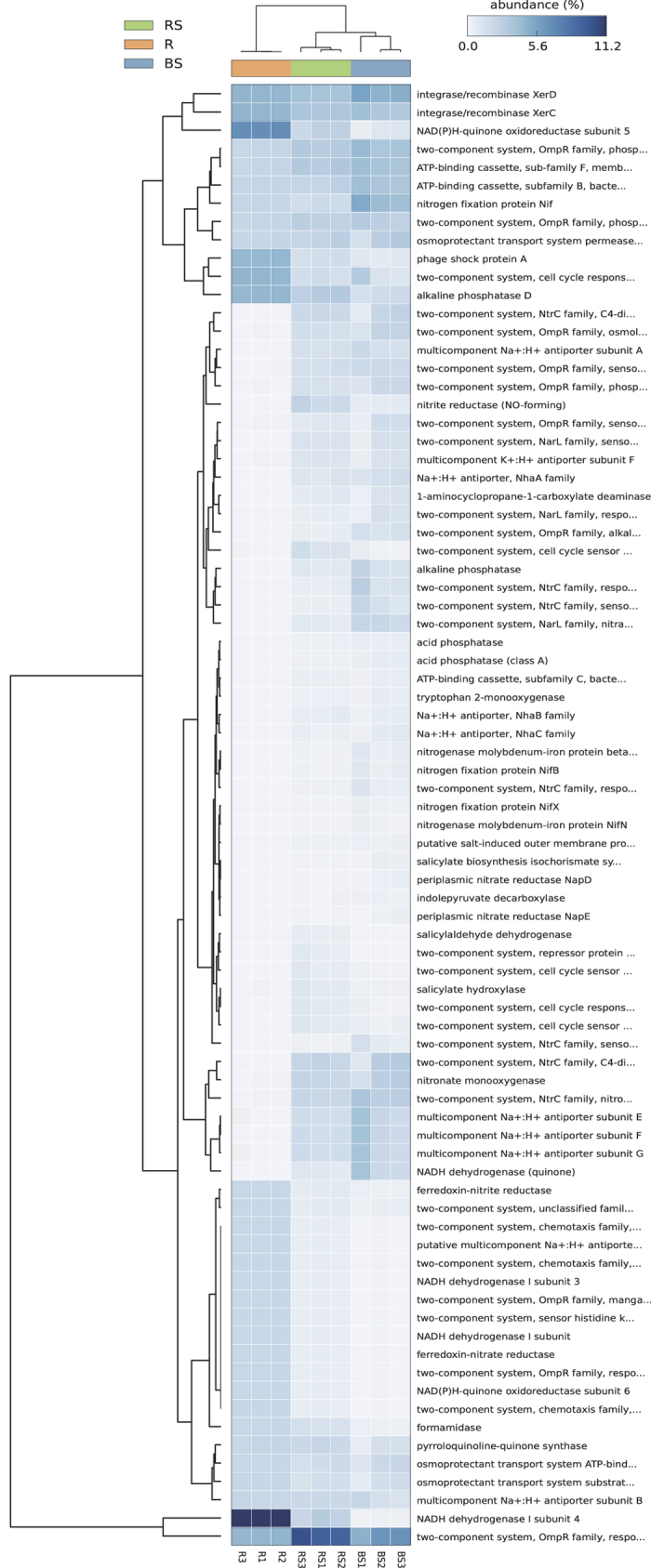
A heatmap showing the hierarchical clustering of the predicted KEGG Orthologs (KOs) functional profiles of bacterial microbiota across all samples. The color bar on top shows the relative abundance of selected genes.

We identified significant differences between the selected functional traits of the three bacterial communities using the level 1 KEGG Orthologs (KOs) function predictions. A heat map reflecting hierarchical clustering of samples and relative abundances of their associated functional potentials is presented in Fig. 3. Relative abundances of each type of functional gene ranged from 10.8% to <1.0%. The vast majority of identified functional profiles (KOs) were shared within the three habitats and encompassed the gene categories related to salt stress acclimatization (including ATP-binding cassette, two-component systems, osmoprotectant transport system, phage shock protein and ion antiporters), nutrient acquisitions (e.g., phosphatase, pyrroloquinoline-quinone synthase, nitrogen fixation protein, nitronate monooxygenase, formamidase and nitrite reductase) and competitive root colonization (site-specific recombinase/integrase and NADH dehydrogenase). It seems that most functional gene categories were far more abundant in rhizosphere and endosphere compared to the bulk soil (Bonferroni-corrected ANOVA, p ≤ 0.01). Furthermore, pivotal enzymes for synthesizing or regulating plant hormones including salicylate hydroxylase, 1-aminocyclopropane-1-carboxylate deaminase, tryptophan 2-monooxygenase and indole pyruvate decarboxylase were predicted to be frequent in rhizosphere despite their low abundance (Supplementary Fig. S3).

### Enhancement of the growth and salt tolerance of crop plants infected by a core fungus

**Figure 4:**
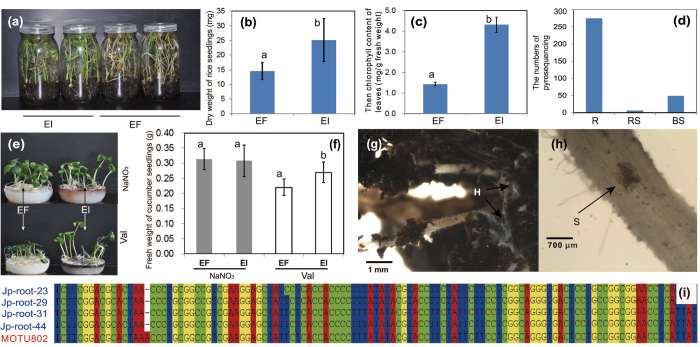
Beneficial effects of a root endophytic Montagnulaceae sp. on plant growth and salt tolerance. (**a**) endophyte-mediated plant (rice seedlings) salt tolerance. (**b**) endophytic colonization on plant biomass accumulation under salt stress conditions. (**c**) endophytic colonization on chlorophyll content of leaves under salt stress conditions. (**d**) the number of pyrosequencing reads of Montagnulaceae sp. detected in the R, RS and BS habitats. (**e,f**) endophytic colonization on plant growth when the nitrogen source occurs in both organic (valine) and inorganic (NaNO_3_) forms in the media. (**g,h**) colonization of Montagnulaceae sp. on root surface (H: hyphae) and aggregation of sclerotium-like (S) structures in root cortical cells. (**i**) alignment of partial ITS1 sequences of Montagnulaceae sp. derived from pyrosequencing data and pure cultures. EI: endophyte-infected; EF: endophyte-free.

The rationale for selection of microbes (either bacteria or fungi) used in this assay is that they were identified as the core microbiota inferred from Venn diagrams (Figs 4d and S4). The strains corresponding to MOTUs identified as Montagnulaceae sp., much more common in the root endosphere than in the bulk or rhizosphere soil, were easily isolated in pure culture (Fig. 4d). The ITS sequences of several Montagnulaceae sp. isolates were highly similar (2 SNPs) to those of the reference MOTU (Fig. 4i). Due to its distinct ITS (GenBank number: KJ125522) sequence divergence from members in the Montagnulaceae family (Pleosporales, Ascomycota), as well as absence of any sporulating structures, this fungus cannot be accurately identified to the genus or species level. We further sequenced the LSU (GenBank number: KJ125523) and RNA polymerase II gene (*rpb2*) (GenBank number: KJ125524) genes of this core fungus and the nucleotide BLAST query, which both supported its inclusion in Montagnulaceae.

We then investigated the phytobeneficial effects of one representative Montagnulaceae sp. isolate Jp-root-44 on two non-host crops (*Cucumis sativus*, cucumber and *Oryza sativa*, rice). When organic nitrogen was present, Jp-root-44 showed a pronounced plant growth promotion effect (t-test, p ≤ 0.05) compared to non-colonized cucumber seedlings (Fig. 4e,f). Salt stress assays demonstrated that endophyte-colonized rice seedlings remained vigorous after irrigation with the 2% sea salt solution (the same as below), while the control plants suffered serious dieback. There were statistically significant differences in seedling dry weights and in chlorophyll content (t-test, p ≤ 0.01) between the treated and control plants (Fig. 4a–c). Microscopic observation strongly supported the supposition that this fungus entered into the inner root tissues of rice and formed microsclerotia-like structures in cortical cells (Fig. 4g,h).

### Bacterial rescue to plant under salt stress

**Figure 5:**
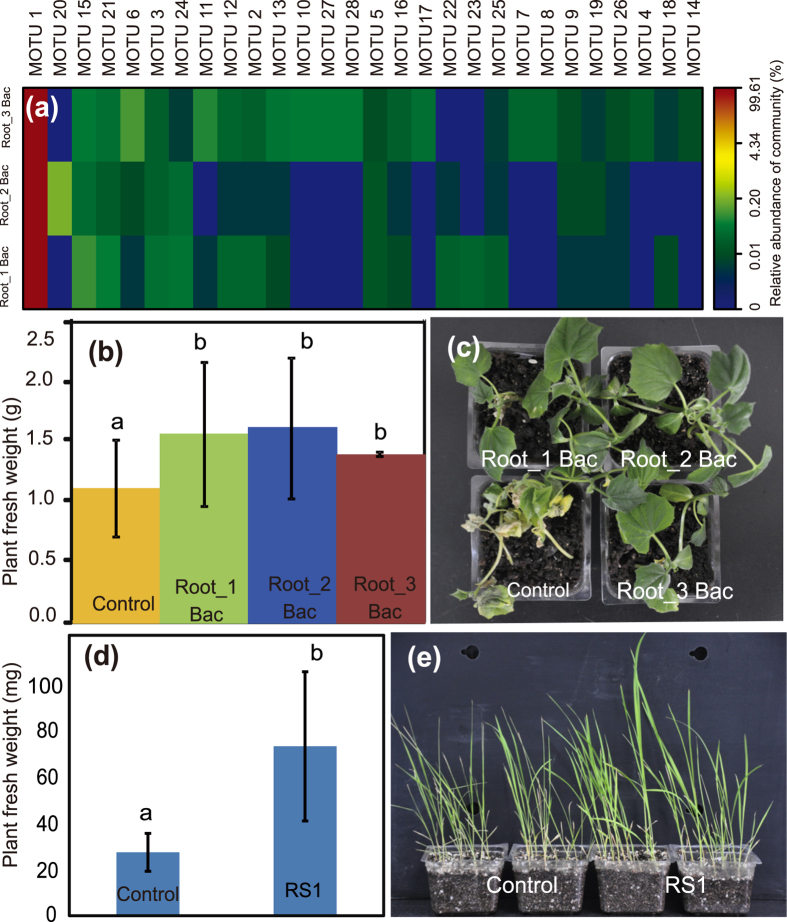
Beneficial effects of a single bacterial culture and a mixed bacterial cultures (MBC) on plant growth and salt tolerance. (**a**) bacterial composition of three replicated MBC using a pyrosequencing approach. (**b,c**) MBC-mediated plant growth and salt tolerance (cucumber seedlings). (**d,e**) a rhizospheric strain, *Pseudomonas sp*. RS1, rescued rice seedlings when exposed to salinity stress.

We further observed the effects of both a core rhizospheric bacterial isolate RS1 (*Pseudomonadales*) and a mixed bacterial culture (MBC) from root endosphere on plant vigor and survival under salt stress conditions (Fig. 5). This isolate was preliminarily identified as *Pseudomonas* sp. (GenBank number: KR817912), as its 16S rRNA gene sequence shared a high similarity (99%) with currently described *Pseudomonas* species. It showed pronounced enhancement in rice development under irrigation with salt water (t-test, p ≤ 0.002), while the leaf edges of control seedlings started rolling inward salt water irrigation (Fig. 5d,e). Similarly, these experiments also demonstrated that MBC rescued cucumber seedlings exposed to salinity and the difference in biomass between the control and treated plants reached a significant level (t-test, p ≤ 0.05) (Fig. 5b,c). Pyrosequencing revealed that 28 MOTUs occurred in the MBC, but only one MOTU belonging to *Pseudomonas* sp. heavily dominated the MBC (Fig. 5a). A phylogenetic tree was constructed to show the genetic relationships among the *Pseudomonas* groups identified from the pure, mixed cultures and pyrosequencing reads from amplicon libraries (Supplementary Fig. S5), indicating a relatively high genetic diversity (a total of 16 distinctive sequences). Unexpectedly, *Acinetobacter* (*Pseudomonadales*) as the dominant bacterial genus in roots, inferred from the amplicon pyrosequencing on root samples (Supplementary Fig. S1), was not found in the MBC.

## Discussion

To date, we continue creating a comprehensive catalogue of microbial species thriving in the rhizo- and endosphere of such model plants as *Arabidopsis thaliana* (Brassicaceae)^21^,^22^, crops (*Zea mays* maize and rice)^23^,^24^ and industrially relevant trees (*Populus* spp.)^25^,^26^. Still, the ecological functioning of microbial communities associated with plants growing under extreme saline environments remain poorly known^17^. By applying an approach previously emphasized for advancing plant microbiome research^27^,^28^, we first explored the “black box” of the bacterial and fungal communities associated with a halophyte plant and functional associated traits.

In order to describe the belowground fungal communities, we used ITS fungal DNA barcoding, recognizing that such an approach presents some limitations^29^, e.g., i) the occurrence of multiple copies with potential high level of variation within an individual, leading to an overestimation of diversity, ii) ITS region with poor phylogenetic resolution due to homoplasy, iii) suboptimal descriptions of lower fungi (such as Glomeromycota and Chytridiomycota), and iv) a somewhat arbitrary similarity cutoff not specifically linked to interspecific polymorphism. In view of these limitations, we were not able to demonstrate MOTUs corresponding to the lower fungi. Meanwhile, the fungal genera detected from this work also have not proven to show a high level of interspecific and intraspecific variations of ITS sequences based on previous investigations. Therefore, we concluded that drawing conclusions from our data was merited.

In this work, we first quantitatively evaluated the structure of bacterial communities associated with different parts of a *S. salsa* and put it in a phylogenetic framework to yield some clues as to the response of microbial communities in three habitats to saline soil and host environments. A pattern of phylogenetic over-dispersion of α-proteobacteria and γ-proteobacteria communities in the rhizosphere suggests the coexistence of a group of distant relatives likely resulting from competitive interactions^30^,^31^. In contrast, communities in the bulk soil and root endosphere tended to be phylogenetically clustered, indicating the coexistence of closely related relatives likely owing to habitat filtering^32^. It is more likely that interspecific competition plays an overwhelming role in structuring rhizospheric bacterial communities, while both biotic (host genetics) and abiotic (salinity) selection pressures drive the assembly process in the root endosphere. Our findings may, at least in part, support the previous hypothesis that phylogenetic clustering predicts the response of microbial communities to environmental extremes^32^,^33^,^34^. Furthermore, our data thus suggests that the ecological strategies adopted by microorganisms to combat salt stress share some common evolutionary traits.

There also appears to be evidence for the marked phylogenetic redundancy in dominant fungal members at the genus level (Fig. 2; Table 2). It is generally assumed that phylogenetic redundancy may create or reflect functional redundancy^35^. The ecological significance of such phylogenetic or functional redundancy has been well-recognized in microbial ecology^35^,^36^ and redundancy can help in the recovery of community functioning following exotic disturbances.

We further used a cost-effective PICRUSt tool for predicting the bacterial community functions, which may enable us to know how the *S. salsa*-associated microbiome is able to adapt to high salt environments and whether there are some microbial functional traits that correlate with plant phenotype. A set of genes related to bacterial salt stress response were selected for analysis^37^,^38^,^39^,^40^,^41^: we discovered an enrichment of diverse two-component systems and ATP-binding cassette (ABC) transporters that may provide a selective advantage for bacterial adaptation to adverse conditions^42^. In this study, many MOTUs identified corresponded to a great variety of well-known halotolerant bacterial species^43^, which probably supported the above interpretation. However, it remains unclear why the rhizosphere soil, rather than the bulk soil or the root endosphere, preferably recruit such taxa (Fig. 1). PICRUSt analysis also predicted high level of NADH dehydrogenase I in endophytic bacteria, which would contribute to their competitive root colonization^44^. Other endophytic bacterial traits predicted by PICRUSt include phosphate solubilization, auxin synthesis and nitrogen fixation features, all of which have been strongly correlated with plant growth. Admittedly, these results can be viewed as supportive as the analysis by PICRUSt does not provide data on actual gene expression in the sample^45^. The relatively high NSTI scores in root samples indicate not only a degree of uncertainty in the prediction of said gene(s) relevance, but also the novelty of microbes in the endosphere. It should be noted that this analysis is restricted to prokaryotic communities only.

To know which portion of the plant phenotype is related to the specialized rhizosphere microbiome, some lines of experimental evidence were presented. We used the core bacterial and fungal members, as well as a MBC, as inocula to illustrate their functional extensions on non-host plants. Results from the analysis of membership and phylogeny-based Venn diagrams (Supplementary Fig. S4), together with the identification of a beneficial bacterial strain and composition of the MBC, produced unambiguous evidence that *Pseudomonas* was of particular importance for plant growth and salt tolerance. The genus *Pseudomonas* is one of the best-studied root beneficial microbes^46^. In the present study, both rhizospheric and endophytic *Pseudomonas* spp. were observed to be responsible for strengthening plant growth and salt stress responses.

Previous field work studies suggest that application of individual inocula/strains often failed to achieve expected outcomes, largely due to competition with native soil microbial communities and limited colonization efficiency^47^. Several researchers recently proposed a novel strategy: to re-construct and transfer the functional microbial groups but not the individual isolates to plants^4^,^48^,^49^. Thus, considerable attention should be devoted to the culturable microbiome, as current inoculum production technology heavily relies on culturability of microbes^6^,^48^. In a recent study, a synthetic bacterial community was constructed by mixing several single strains at the same ratio for inoculation^50^,^51^. Unlike the synthetic community, MBCs generated using a direct liquid fermentation approach would mimic the pattern of the natural bacterial communities, an important feature underlying synthetic microbial ecosystems^52^. Under greenhouse conditions, however, there was no clear difference between the use of an individual strain and the use of the MBC with respect to beneficial effects on plants. A variety of methods to enrich diverse rhizobacteria groups were developed^53^, enabling us to further facilitate the design of microbial consortia^54^. No doubt future attempts upon refinement of this technology will merit positive results.

Regarding core culturable fungal members, we paid particular attention to the melanized fungus, an unidentified Montagnulaceae sp. Inoculation tests showed that it would behave as a plant growth promoter under low levels of organic nitrogen. Thus, it appears that this fungus could be aiding the plant in increasing degradation of organic proteins and enhancing their availability for absorption by the plant roots. Enhanced organic nitrogen scavenging was seen previously in studies involving rhizobacteria^55^. Microscelerotia-like structures formed in root tissues confirmed not only the nature of its endophytism but also its classification as a dark septate endophyte (DSE). The positive effects of DSEs on plant growth and nutrition acquisition have been implicated through the use of a meta-analysis approach^56^. Montagnulaceae sp. further displays the ability to increase plant salt tolerance. The mechanism of DSEs-mediated plant salt response, however, is poorly understood, although it has often been hypothesized that the high melanin levels in hyphae are responsible for alleviating host abiotic stresses^57^. Collectively, there is a growing consensus recognizing that DSE symbiosis may play a much more important role on an ecologic and evolutionary level than previously viewed^18^,^58^.

**Figure 6:**
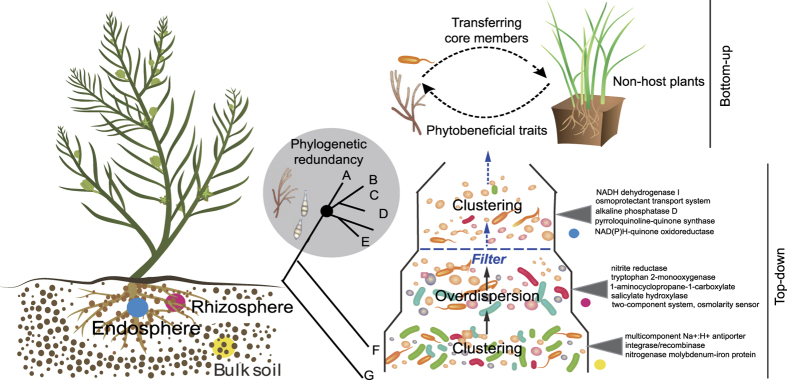
Conceptual illustration of the microbial community assembly and functional traits in the bulk soil (BS), rhizosphere soil (RS) and root endosphere (R). A rapid loss of diversity from soil (pedoshere) to the root endosphere (phytosphere) indicates the presence of a biotic filter at the root-soil interface (e.g., host genotype and root exudates). The dominant α-proteobacteria and γ-proteobacteria communities in BS and R showed patterns of phylogenetical clustering, suggesting the microbial response to environmental extremes; there were over-dispersion trends in RS. In all habitats, a group of closely related MOTUs assigned to the same microbial lineages (at the genus level) indicated some degree of phylogenetic redundancy, a known buffering mechanism against stresses. The representative abundant functional genes predicted by PICRUSt in each habitat were presented. These genes were often related to bacterial salinity tolerance and improved plant performance. Re-colonization and salt stress assays (a bottom-up approach) confirmed the extended phytobeneficial traits of some core culturable members on non-host plants.

In conclusion, our study gains novel insights into the structure and functional profiling in a halophyte rhizosphere microbiome. It can be seen that an ecological patterned root-microbial interaction strategy has been adopted in *S. salsa* system to confront soil salinity. We particularly argue that the integration of top-down (pyrosequencing-based microbial community survey), as well as bottom-up (recolonization-based experimental assay) approaches will be required to gain a better understanding of microbial functional traits (Fig. 6). There is also evidence that the root beneficial symbionts may be predicted by defining the core microbiota. This is a proof-of-principle study and the universality of this phenomenon across different plant species and environments remains to be determined. We still believe that such information is valuable for capitalizing on the culturable microbiome for field applications and accelerating the design of synthetic or natural microbial communities that can benefit salt-susceptible plants. In addition, special considerations should be given to the tripartite “plant-fungal-bacterial” symbioses that potentially generate synergistic effects on plants. Looking forward, these approaches show substantial promises for saline agriculture.

## Methods

### Field sampling

*S. salsa* (Amaranthaceae) is widely distributed in China’s eastern coastal regions. In July 2013, we collected *S. salsa* seedlings (average height of 30 cm) growing in harsh saline soils (Dongying, Shandong province, N 37°23′43”, E 118°55′25”) with 2.67% soluble salt and a pH of 8.51. To avoid potential sampling bias caused by soil heterogeneity, three sampling plots were selected (around 3–5 m apart) at the above location. Three plants per plot were randomly harvested (as replicates from each plot). Therefore, in this study we mainly focus on dissecting the plant belowground microbiome under this defined stress conditions. While they might have an effect on the microbial community structure, the soil (pH, salt gradient and nutrients) and plant features (different development stages) are not considered at the scope of this work.

Samples were grouped into three fractions: bulk soil, rhizosphere soil and root endosphere. Sub-samples of each fraction from one plot were merged into one sample. For the collection of bulk soil, samples were taken from a depth of 20 cm using an auger borer. We collected rhizosphere soil (a region of soil of about 1 mm surrounding roots) using previously described methodology^21^. The loose soil was removed carefully from the root surface and roots containing a thin layer of remaining soil aggregates were transferred into sterile 50-ml tubes (BD Falcon) with 30 ml of a 100 mM phosphate buffer solution (PBS, pH = 7.2). All sample tubes were placed into a cold storage container (10 °C for not more than 24 h) and taken to the lab for further processing. Tubes were vortexed at maximum speed for 20 s, allowing the release of most of the rhizosphere soil. The resulting turbid solutions were centrifuged for 10 min at 5000 g at 4 °C to form a pellet. The pellets were then frozen in liquid nitrogen and stored at −75 °C until use.

The protocol for root surface sterilization to kill epiphytic microbes used in this study proved to be efficient as in our previous studies^59^. To remove possible DNA contamination from dead surface microbial cells, the commercial DNA AWAY™ surface decontaminant (Molecular Bioproducts) was used to completely degrade DNA. The sterilized roots were immersed in DNA AWAY™ solution for 5 min followed by rinsing three times with sterile water.

### DNA extraction

A DNeasy Plant Mini Kit (QIAGEN) was used to extract DNA from plant materials. A Power Soil DNA Isolation Kit (MOBIO) was used for DNA extraction of soil samples according to the manufacturer’s instructions with the following modifications: Since high salt concentrations in bulk soil can interfere with the DNA extraction process, the samples were initially homogenized in sterile 100 mM PBS and then centrifuged (10 min, 5000 g, 4 °C), a process known to remove salt to a great extent from the samples. After the kit solution C1 was added, PowerBead Tubes were placed in a 70 °C water bath for 10 min. The samples were ground in liquid nitrogen using a sterile, frozen mortar and pestle. About 100 mg of frozen plant material powder was used for the extraction. To obtain high yields of DNA, we processed five subsamples in parallel and used 120 μl of washing buffer to elute all of the collection tubes twice during the final step of the kit protocol.

### PCR amplification and amplicon sequencing

A total of 32 amplicon libraries were constructed for the 454-pyrosequencing. We used two primer sets to target either the bacterial 16S rRNA gene or the fungal internal transcribed spacer 1 and 2 (ITS1 and ITS2) of the rRNA gene cluster. A series of primer pairs are available for amplifying different variable regions of 16S rRNA in bacteria. Of these, the 799F-1193R primer (spanning the V3-V4 variable regions) proved useful to reduce the occurrence of amplification of contaminating plant-derived sequences^60^. Primers spanning the V5-V7 regions (356F-1064R primers) were developed to capture more diverse bacterial domains^61^. Touchdown PCR was used to further minimize host rRNA gene amplification^22^. The ITS1F-ITS2 (ITS1 region) and the fITS7-ITS4 (ITS2 region) primer pairs were used for fungal ITS amplification^62^,^63^,^64^. We developed a nested PCR method to efficiently reduce plant ITS sequence contamination^65^. The respective protocol and detailed determination of specificity of fungal primers were provided in Supplementary Experimental Procedures.

Amplicons were pooled, gel-purified and sequenced with the 454 GS FLX+platform (Roche) located at the Beijing Genomics Institute (Shenzhen, China). Fungal and bacterial amplicon libraries were clonally amplified using emulsion PCR. The forward primer comprised 454 fusion primer adaptor A, a 4-bp key sequence (TCAG), a 10-bp multiplex identifier (MID) barcode and the specific primer sequence, whereas the reverse primer comprised adaptor B, a 4-bp key sequence and the specific primer sequence (Supplementary Table S1).

### Pyrosequencing and data analyses

Multiple levels of quality control were applied during sequencing and data analysis. Sequence quality control was performed using Mothur (v1.31.2)^66^. The trim.seqs command was used to trim the sequence when the average quality score over a 50 bp window dropped below 25. Sequences with ambiguous nucleotides, homopolymers of more than 8 bases, or less than 200 bp (for fungi, shorter than 100 bp) in length were removed. The unique.seqs. command implemented in Mothur was used to obtain a non-redundant set of sequences. Chimeras were identified and removed using UCHIME (v4.2, http://drive5.com/uchime). The optimized reads were taxonomically assigned using an RDP classifier (a naïve Bayesian classifier) with a bootstrap cut-off of 80%^67^. Operational taxonomic units (MOTUs) with a 97% similarity threshold were defined using the average neighbor clustering algorithm implemented in Mothur. All bacterial MOTUs annotated as plant plastids or mitochondrial 16S rRNA sequences were removed from the MOTU table. Representative sequences (the most abundant sequence from each MOTU) were aligned to the SILVA Reference Alignment using the NAST algorithm and then trimmed to a common alignment region with an average length of 260 bp^68^. To assign taxonomic affiliations to the sequences obtained, we conducted BLAST-based similarity searches against the SILVA database.

For fungi, non-chimeric MOTUs were clustered in UCLUST at a 97% pairwise similarity cutoff using the UNITE database. The pyrosequencing reads were analyzed using sequence similarity searches against the UNITE fungal ITS database. Sequence alignment for ITS1 and ITS2 was generated in MUSCLE v3.8 using default parameters^69^. The remaining conserved SSU, 5.8S and LSU fragments were not removed, as they would not have influenced the taxonomy of the MOTUs. Fungal singletons (MOTUs containing only one sequence read) were excluded from further analysis.

### Statistical analyses

All *sff* (Standard Flowgram Format) files representing raw pyrosequencing reads were deposited with the European Nucleotide Archive (ENA) under study accession number: PRJEB8726 (www.ebi.ac.uk/ena/data/view/PRJEB8726). To test for sampling effectiveness, rarefaction curves based on the resulting OTU tables rarefied to the lowest numbers of reads obtained for any single sample, were generated using the vegan R package (v2.15.3) (Supplementary Fig. S6).

MEGA v5.2 and DnaSP v.5.0 were used to calculate the mean p-distance for estimating the infrageneric genetic diversity of the dominant fungal lineages^70^. Meanwhile, the phylogenetic diversity and structure of the dominant bacterial (α-proteobacteria and γ-proteobacteria) communities were quantitatively assessed with Phylocom v4.2^71^. Phylogenetic trees based on maximum likelihood (ML) were constructed using the RAxML BlackBox program (http://embnet.vital-it.ch/raxml-bb/) with the gamma-partitioned model and trees were edited using FigTree v1.4.2. Faith’s index of phylogenetic diversity^72^, the Net Relatedness Index (NRI), the Nearest Taxon Index (NTI)^73^ and Rao’s quadratic entropy were measured to test for clustered^74^, over-dispersed or random patterns in the bacterial communities. Ultrametric trees were generated for the 16S rRNA data sets in BEAST v1.8.2. MCMC (Markov chain Monte Carlo); runs of 100,000,000 generations were needed to achieve ESS (effective sample size) values >200. Because of an absence of temporal calibration, a strict molecular clock scaled to substitutions was used^75^. Convergence of the two chains was checked using Tracer (http://beast.bio.ed.ac.uk/Tracer). Maximum credibility trees were generated in TreeAnnotator (in the BEAST package). Positive NRI/NTI values indicate phylogenetic clustering; negative values indicate phylogenetic over-dispersion; values close to zero indicate random phylogenetic structure. The statistical significance of NRI and NTI was assessed based on comparison of phylogenetic distance observed and that of 999 permutations of a null model.

In order to avoid the major limitation of the shotgun metagenomics analysis of endophytic microbial communities owing to a large amount of host DNA^76^, in this work, we utilized the PICRUSt: Phylogenetic Investigation of Communities by Reconstruction of Unobserved States (http://picrust.github.io/picrust/). This software allows inference of the functional profile of a microbial community based on marker gene survey along the samples^77^. PICRUSt uses a set of known sequenced genomes and user-provided counts for 16S rRNA MOTUs, representing the marker gene sequences accompanied with its relative abundance in each of the samples. It provides a functional-gene-count matrix, containing the count of each functional-gene in each of the samples surveyed. Thus the estimation of the approximate content of functional genes in an unknown genome represented by a 16S rRNA phylotype in the user samples is based on such values for the phylogenetically closest known sequenced genome or genomes^77^. The online galaxy version (http://huttenhower.sph.harvard.edu/galaxy/, v1.0.0) was used for analysis. The obtained MOTUs tables were subsequently normalized by the predicted 16S rRNA copy number and metagenomes were predicted from the Kyoto Encyclopedia of Genes and Genomes (KEGG) catalogue. We obtained all KEGG Orthologs (KOs) annotations and a set of *KOs* involved in salt adaptation, nutrient solubilization and root-bacterial interactions were selected. Regarding the accuracy of metagenome predictions, the Nearest Sequenced Taxon Index (NSTI, implemented in the PICRUSt), which evaluating the evolutionary distance between each MOTU in a sample to its closest reference genome, were pre-calculated. Statistical analysis, visualization and edition the final predicted metagenome were performed using STAMP software (Statistical Analysis of Metagenomic Profiles, v2.1.3)^78^.

### Plant and microbial materials for inoculation

To provide direct evidence of the phytobeneficial traits of the culturable microbiome on plant performance, culture-dependent method was employed to recover endophytic fungi and rhizospheric bacteria *S. salsa*. Briefly, the surface disinfected root fragments (5 mm in length) were put on 2% malt extract agar (MEA, Oxoid) for incubation in darkness at 20 °C. The emerging hyphae from the ends of root fragments were cut and transferred to potato dextrose agar (PDA) for purification. As described above, 1 g of rhzisopshere soil sample was re-suspended in 99 ml PBS and a series of 10-fold dilutions were made from the suspension. Dilution of 10^−5^ was used to isolate the rhizospheric bacteria. 100 μl of diluted suspension was spread evenly onto *Pseudomonas* isolation agar (PIA, BD Difco) and 1/10 tryptic soy agar (TSA) plates. The colonies with different appearance and characters were picked up and purified by streaking on LB medium (10 g tryptone, 5 g yeast extract, 5 g NaCl, 15 g agar, and 1 L water).

The Montagnulaceae sp. isolated from roots was used as fungal inoculum. The rhizospheric bacterial isolate RS1 was cultured to an optical density (OD) at 600 nm of 1.0 in liquid nutrient broth-yeast extract medium (NBY). Composition per liter: nutrient broth (dehydrated) 8.0 g, yeast extract 2.0 g, K_2_HPO_4_ 2.0 g, KH_2_PO_4_ 0.5 g, Glucose 5.0 g and 1 ml 1 M MgSO_4_·7H_2_O. Beyond the pure cultures, we further generated a mixed bacterial culture (MBC) that originated from the root endosphere for inoculation. In brief, surface sterilized root tissues were ground with a mortar and pestle in 0.1 M PBS buffer to obtain a mixed suspension. After settling for 5 min, 1 ml of this suspension was transferred to tryptic soy broth (TSB) and diluted 1:10 to support the growth of a wide variety of bacteria. Imazalil sulfate (Sigma) was added to the TSB (50 μg ml^−1^) to prevent fungal growth. The suspensions were incubated in a shaker (28 °C and 180 rpm min^−1^) until OD = 1.0. The composition of the mixed bacterial culture was also determined using a pyrosequencing approach. For inoculation, either single or mixed culture was harvested by centrifugation at 5000 g for 5 min and the bacterial pellet was re-suspended and diluted to OD of 0.3. Due to the fact that some of the important traits might be localized on bacterial plasmids, which would be lost during the culturing, we kept the selection pressure by adding the sea salt into the media to a final concentration of 2% (w/v). Rice (*Oryza sativa*) and cucumber (*Cucumis sativus*) seeds were thoroughly disinfected and germinated on 1/5 Murashige and Skoog (MS) medium to generate gnotobiotic seedlings.

### Plant growth and salt stress assays

We followed a previously described method and used peat moss: perlite: vermiculite (2:1:1) as a plant growth substrate^79^. As the fungus did not sporulate, fresh mycelia were mixed with sterile water (5 mg ml^−1^) for inoculation. For bacterial inoculation, roots were immersed into cell suspensions for at least 1 h and then planted. For the salt stress experiment, a 2% (w/v) sea salt solution was used to treat pre-colonized and non-colonized control plants (15 ml per seedling) after 10 days. Sterile water lacking fungal or bacterial cells was used as the control treatment. Three to five replicates for each treatment were performed. Two-sample t-tests were used to evaluate any differences between the treatment and the control samples.

## Additional Information

**How to cite this article**: Yuan, Z. *et al*. Specialized Microbiome of a Halophyte and its Role in Helping Non-Host Plants to Withstand Salinity. *Sci. Rep*. **6**, 32467; doi: 10.1038/srep32467 (2016).

## Supplementary Material

Supplementary Information

## Figures and Tables

**Table 1:** Phylogenetic diversity and structure of α-proteobacteria and γ-proteobacteria belowground communities in terms of Faith’s phylogenetic diversity (PD), Rao’s quadratic entropy (QE), Net Relatedness Index (NRI) and Nearest Taxon Index (NTI).

	α-proteobacteria			γ-proteobacteria		
	R	RS	BS	R	RS	BS
PD	0.668	2.265	2.644	0.229	4.516	2.707
QE	0.057	0.060	0.074	0.031	0.103	0.061
NRI	**4.201**	−0.645	**3.158**	0.494	**−2.690**	**8.789**
NRI P-value	**0.008**	0.273	**0.001**	0.319	**0.001**	**0.000**
NTI	1.360	−0.911	−0.178	1.383	−0.097	**2.995**
NTI P-value	0.092	0.179	0.443	0.103	0.481	**0.003**

NRI and NTI are indicators of phylogenetic clustering or phylogenetic overdispersal inferred using PHYLOCOM. All indices are based on ultrametric trees constructed with BEAST program. More details are presented in main text. *P-value based on comparison of phylogenetic distance observed and that based on a 999 permutations of a null model. R = root endosphere, RS = rhizosphere soil, and BS = bulk soil.

**Table 2:** DNA polymorphism of phylogenetically redundant fungal lineages based on ITS1 or ITS2 sequence analysis.

	Beauveria^a^	Retroconis^b^	Leptosphaeria^b^	Monosporascus^a^
No. of OTUs	24	11	23	18
No. of polymorphic sites	14	16	19	18
Mean p-distance	0.012	0.042	0.023	0.025
π (nt diversity)	0.0115 ± 0.0012	0.0399 ± 0.0119	0.0223 ± 0.0051	0.0230 ± 0.0081

Infrageneric diversity was estimated based on the calculation of p-distance and nucleotide diversity (π).

Notes: “a” and “b” implied that the data was from ITS1 and ITS2 pyrosequencing reads, respectively.
