# Supplementary material for: Specialized Microbiome of a Halophyte and its Role in Helping Non-Host Plants to Withstand Salinity
Source: Sci Rep. 2016 Aug 30;6:32467. doi: 10.1038/srep32467 (PMC5004162; doi:10.1038/srep32467)
Supplement: Supplementary Information [file srep32467-s1.doc]

**Supplementary Information**

**Specialized Microbiome of a Halophyte and its Role in Helping Non-host Plants to Withstand Salinity**

Zhilin Yuan^1,#^, *, Irina S. Druzhinina^2,#^, Jessy Labbé^3^, *, Regina Redman^4^, Yuan Qin^1^, Russell Rodriguez^4, 5^, Chulong Zhang^6^, Gerald A. Tuskan^3^ and Fucheng Lin^6^

**Contents**

1. Supplementary Experimental Procedures
2. Supplementary Results
3. Supplementary Figures and Tables
4. References

**1. Supplementary Experimental Procedures**

*1.1* *Determining specificity of fungal primers*

Primer pair specificity to fungal ITS from the root samples was tested as follows^1^: the purified PCR products were ligated into the pGEM-T Easy vector and transformed into *Escherichia coli* JM109 competent cells and sequenced as a clone library. Unfortunately, both the ITS1F-ITS2 and the ITS3-ITS4 primer pairs exclusively amplified the host ITS sequences (34 and 22 positive clones were randomly selected from ITS1 and ITS2 libraries, respectively for Sanger sequencing) as confirmed by sequence similarity search (blastn) against NCBI database. The average length of ITS1 or ITS2 in S. salsa was 230 bp. Nested PCR methods were thus employed to reduce nonspecific amplification. The first amplification was initiated using the NSA3-NLC2 primer pair (amplifying the entire ITS region together with a small region of 18S and 28S rDNA), which is considered to be Dikarya-specific^2^. The second amplification was performed using two primer sets (ITS1F-ITS2 and fITS7-ITS4) to amplify the ITS1 and ITS2 regions, respectively. The specificity of desirable PCR product was also evaluated again as mentioned above. All of the sequences obtained from the 12 selected positive clones corresponded to fungal lineages.

**2. Supplementary Results**

*2.1 Identifying the core microbiota in S. salsa*

We used both membership and phylogeny-based Venn diagrams to identify the core bacterial colonizers^3^. The membership-based Venn diagram showed that 15 MOTUs were shared across all samples. 6 lineage MOTUs overlapped when using a phylogeney-based approach. Each lineage MOTU contained up to four taxonomic MOTUs. Most core MOTUs identified were present when using both of the approaches.These core members were clustered in three bacterial classes: Sphingobacteria, α-Proteobacteria, and γ-Proteobacteria. In particular, a remarkable abundance of lineage 1 MOTUs belonging to *Pseudomonas* was recorded, followed by lineage 3 MOTUs that consisted of two well-known halo-bacterial genera: *Marinobacter* and *Marinimicrobium*^4,5^.

For the fungal microbiome, we contend that it is not rational to construct phylogenies across a wide range of fungal taxa, owing to the high degree of homoplasy in ITS (in particular for ITS1) sequences^6^.We thus used only membership-based Venn diagrams to identify core fungal community members. A total of 36 MOTUs were shared among the three sample types. Most of these belonged to the genera *Monosporascus* (Xylariales), Montagnulacea sp. (Pleosporales), *Paraphaeosphaeria* (Pleosporales), and *Alternaria* (Pleosporales); the remaining MOTUs were not able to be unclassified.

1. **Supplementary Figures and Tables**

**
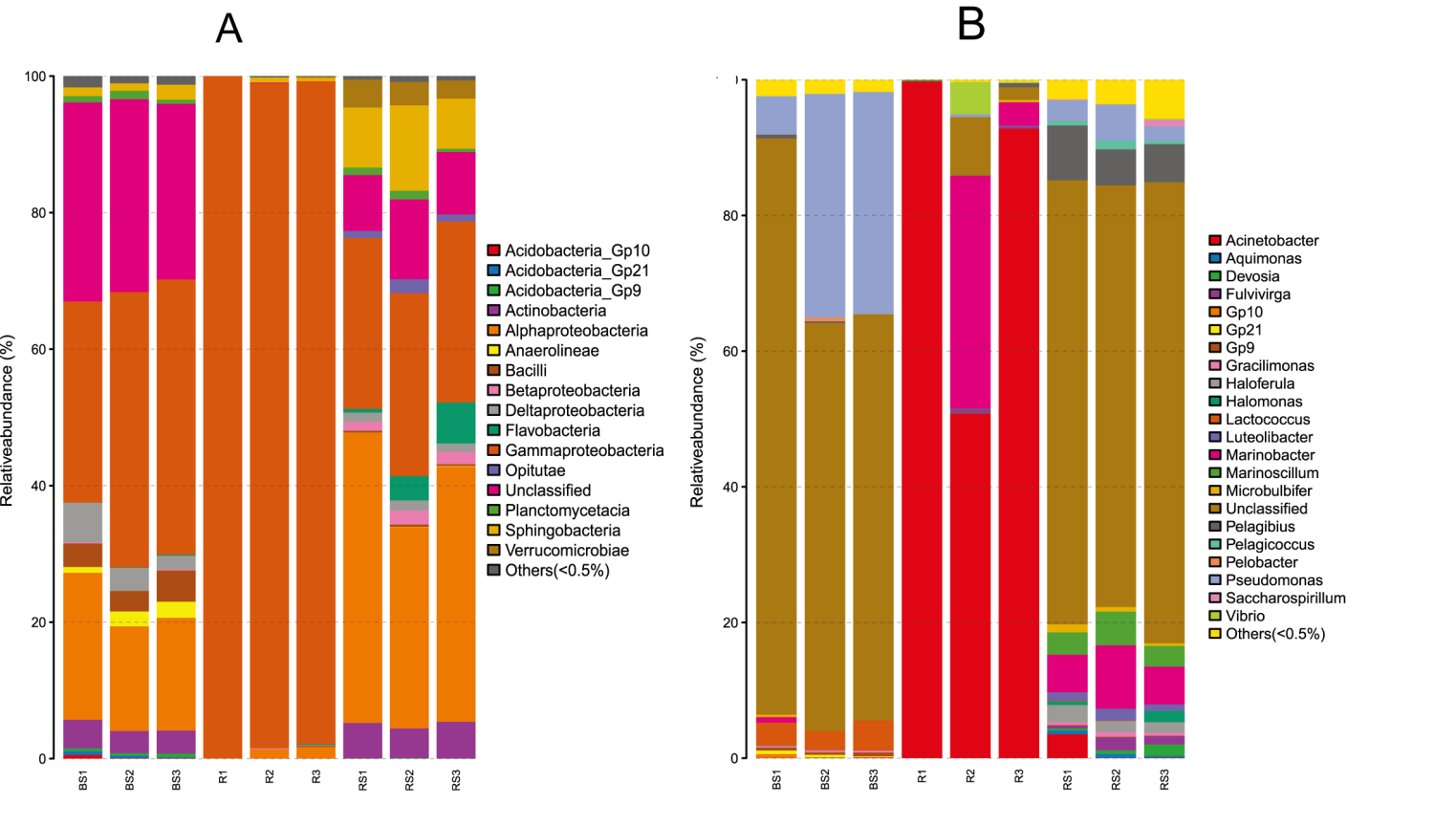
**

**Fig. S1** Relative abundance of bacterial communities in the R, RS, and BS habitats represented by bar charts. A: at the class level; B: at the genus level.

**
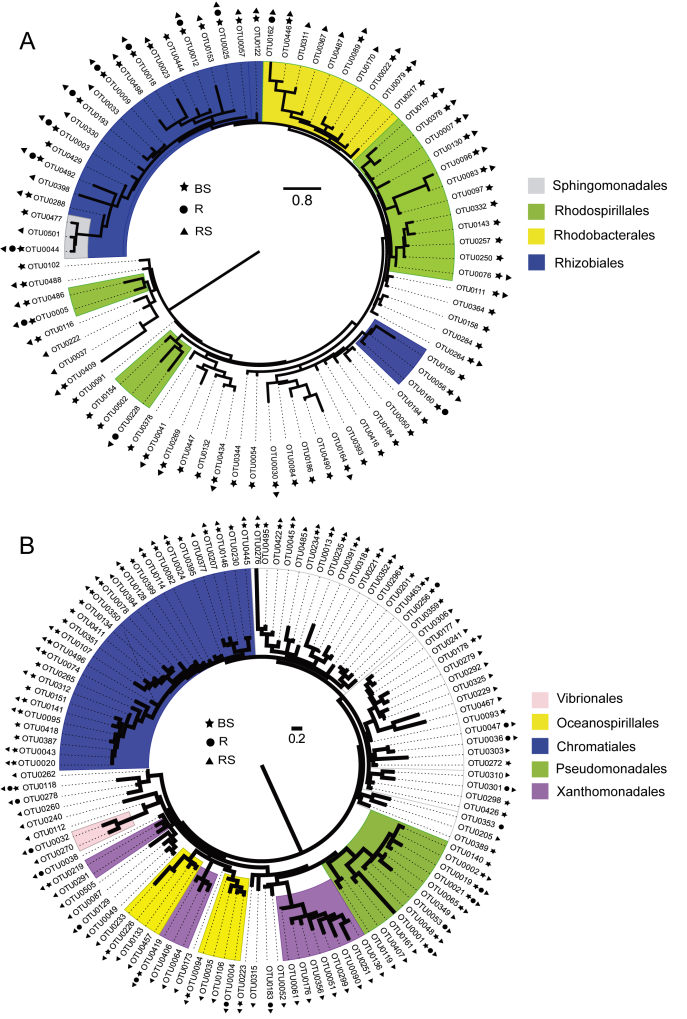
**

**Fig. S2** Maximum-likelihood phylogenetic trees of α-Proteobacteria (A) and γ-Proteobacteria (B) based on 16S rRNA gene sequences. The major orders of the above classes are indicated with different colors; the remaining branches represent the unclassified members. The origin of MOTUs is indicated by symbols close to each MOTU number.


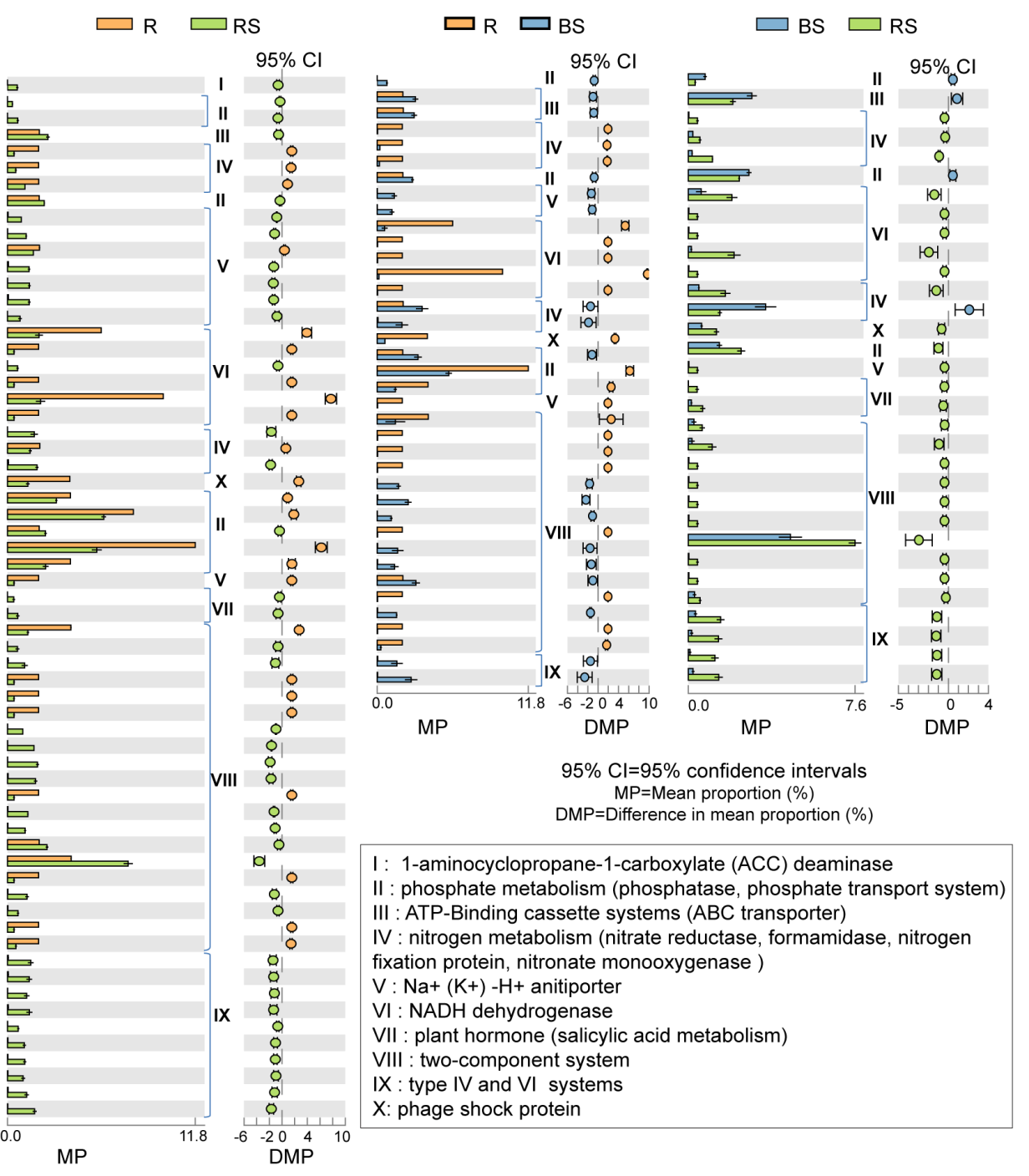


**Fig. S3** Detailed comparisons of the metagenomic differences between two groups. The relative abundances of functional genes were analyzed using STAMP. Extended error bars and 95% confidence intervals were presented. Two-side Welch's t-test and Benjamini-Hochberg FDR correction were used in two-group analysis. All the differences reached a significant level (Bonferroni-corrected P<0.05).


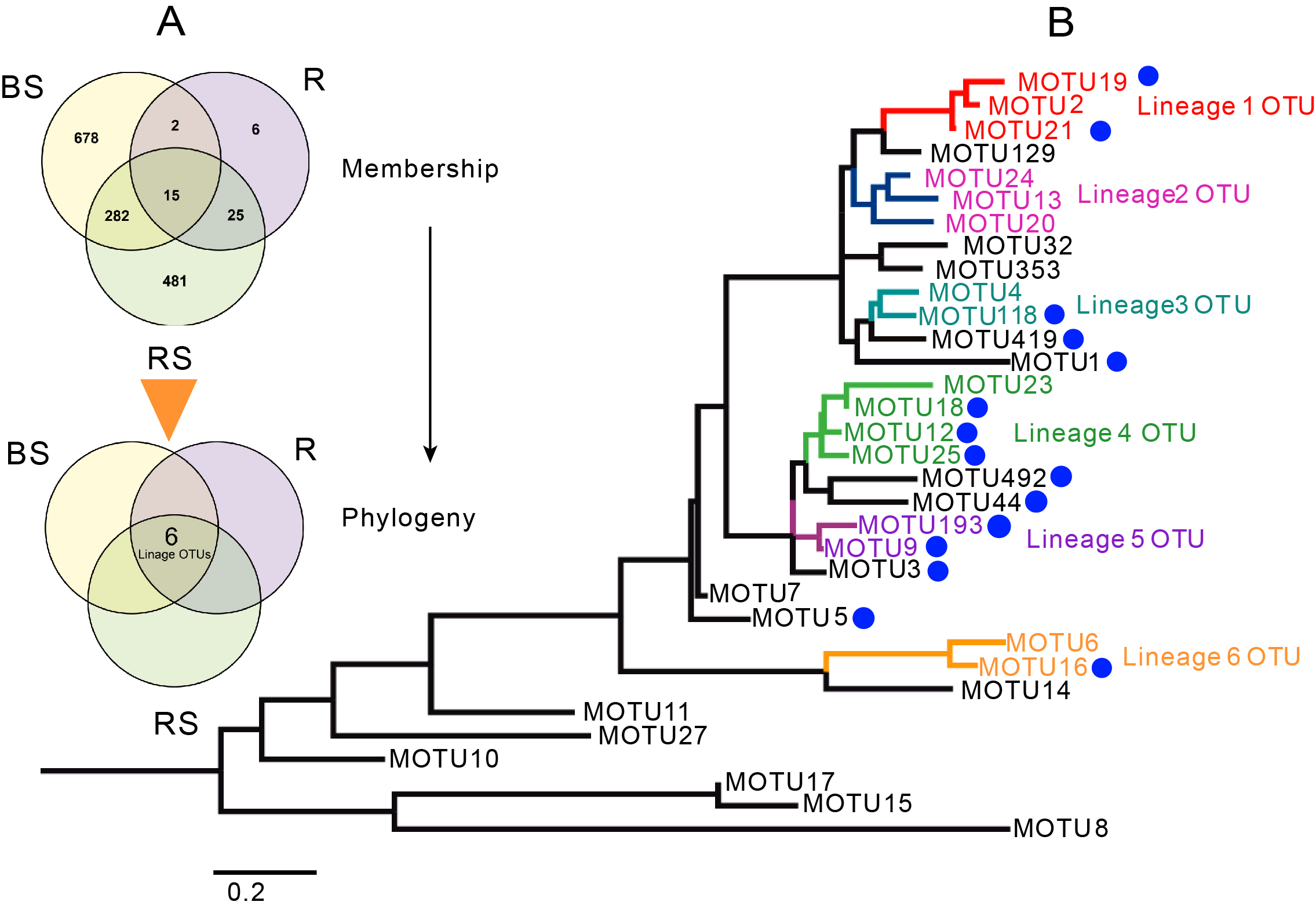


**Fig. S4** The core bacterial microbiota in the belowground microbiome and their phylogenetic relatedness. A: 15 MOTUs shared by the root endosphere, rhizosphere soil, and bulk soil samples based on the phylogeny-based Venn diagram; 6 lineage MOTUs overlapped based on the membership-based Venn diagram. B: phylogeny of core bacterial MOTUs and some close relatives based on analysis of 16S rDNA genes. Blue solid circles indicate the core MOTUs detected with the membership-based Venn diagram.


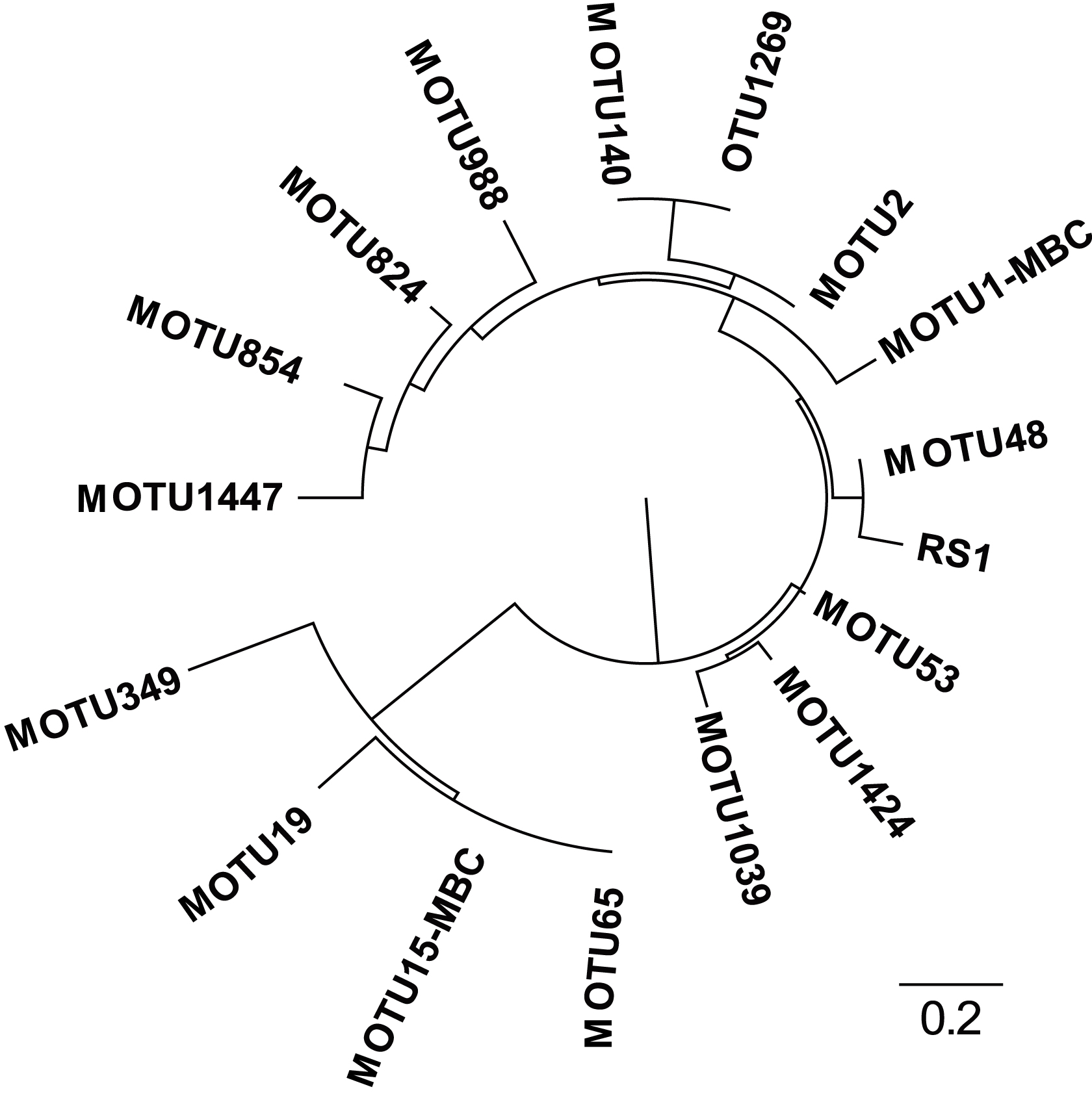


**Fig. S5** Phylogeny of Pseudomonas spp. based on their 16S rRNA sequences obtained from the pure culture (RS1), the mixed bacterial culture (MBC) and pyrosequencing reads from amplicon libraries.

**
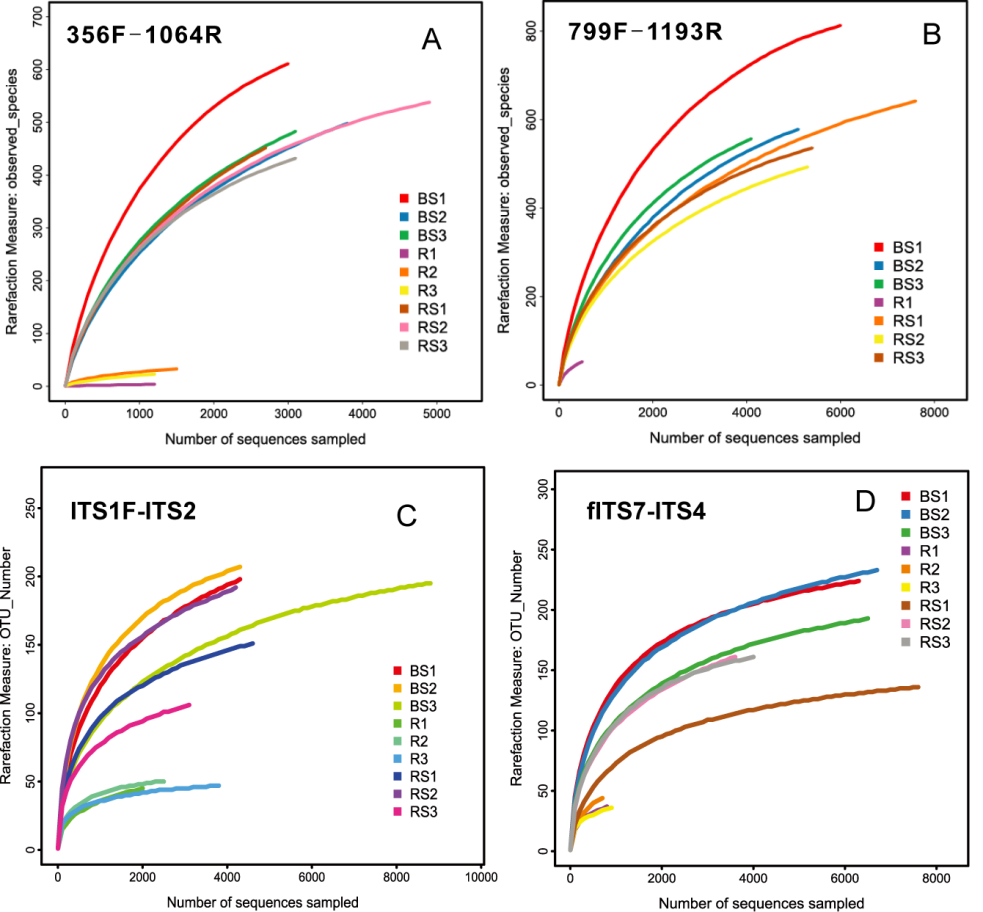
**

**Fig. S6** Rarefaction analyses of the bacterial (A, B) and fungal libraries (C, D) of *S. salsa*. Each curve illustrates the cumulative number of operational taxonomic units (MOTUs) at a phylogenetic distance of 0.03. The replicated libraries from each type of sample are indicated by “1”, “2” and “3”. BS=bulk soil, RS=rhizosphere soils, R=root endosphere.

**Table S1** Fusion primers with Roche/454 adapters, MIDs, and four base library key sequences used for library construction.

**4. References**

1. Yuan, Z. L., Zhang, C. L., Lin, F. C. & Kubicek, C. P. Identity, diversity, and molecular phylogeny of the endophytic mycobiota in rare wild rice roots (*Oryza granulata*) from a nature reserve in Yunnan, China. *Appl. Environ. Microbiol.* **76**, 1642-1652 (2010).

2. Martin, K. J. & Rygiewicz, P. T. Fungal-specific PCR primers developed for analysis of the ITS region of environmental DNA extracts. *BMC Microbiol.* **5**, 28 (2005).

3. Shade, A. & Handelsman, J. Beyond the Venn diagram: the hunt for a core microbiome. *Environ. Microbiol.* **14**, 4-12 (2012).

4. Romanenko, L. A. et al. *Marinobacter bryozoorum* sp. nov. and *Marinobacter sediminum* sp. nov., novel bacteria from the marine environment. *Int. J. Syst. Evol. Microbiol*. **55**, 143-148 (2005).

5. Lim, J. M. et al. *Marinimicrobium koreense* gen. nov., sp. nov. and *Marinimicrobium agarilyticum* sp. nov., novel moderately halotolerant bacteria isolated from tidal flat sediment in Korea. *Int. J. Syst. Evol. Microbiol*. **56**, 653-657 (2006).

6. Alvarez, I. & Wendel, J. F. Ribosomal ITS sequences and plant phylogenetic inference. *Mol. Phylogenet. Evol*. **29**, 417-434 (2003).
